# Parental segregation study reveals rare benign and likely benign variants in a Brazilian cohort of rare diseases

**DOI:** 10.1038/s41598-022-11932-z

**Published:** 2022-05-11

**Authors:** Caio Robledo D.’Angioli Costa Quaio, Jose Ricardo Magliocco Ceroni, Murilo Castro Cervato, Helena Strelow Thurow, Caroline Monaco Moreira, Ana Carolina Gomes Trindade, Cintia Reys Furuzawa, Rafaela Rogerio Floriano de Souza, Sandro Felix Perazzio, Aurelio Pimenta Dutra, Christine Hsiaoyun Chung, Chong Ae Kim

**Affiliations:** 1 Instituto da Criança (Children’s Hospital), Hospital das Clínicas HCFMUSP, Faculdade de Medicina FMUSP, Universidade de São Paulo, São Paulo, SP Brazil; 2 Fleury Medicina E Saúde, São Paulo, SP Brazil; 3 Laboratório Clínico, Hospital Israelita Albert Einstein, São Paulo, SP Brazil; 4 Division of Rheumatology, Universidade Federal de Sao Paulo, Sao Paulo, Brazil; 5 Instituto da Criança do Hospital das Clínicas da FMUSP – Unidade de Genética, Av. Dr. Enéas Carvalho de Aguiar, 647. Cerqueira César, São Paulo, SP CEP: 05403-900 Brazil

**Keywords:** Clinical genetics, Genomics, Medical genetics, Mutation

## Abstract

Genomic studies may generate massive amounts of data, bringing interpretation challenges. Efforts for the differentiation of benign and pathogenic variants gain importance. In this article, we used segregation analysis and other molecular data to reclassify to benign or likely benign several rare clinically curated variants of autosomal dominant inheritance from a cohort of 500 Brazilian patients with rare diseases. This study included only symptomatic patients who had undergone molecular investigation with exome sequencing for suspected diseases of genetic etiology. Variants clinically suspected as the causative etiology and harbored by genes associated with highly-penetrant conditions of autosomal dominant inheritance underwent Sanger confirmation in the proband and inheritance pattern determination because a “de novo” event was expected. Among all 327 variants studied, 321 variants were inherited from asymptomatic parents. Considering segregation analysis, we have reclassified 51 rare variants as benign and 211 as likely benign. In our study, the inheritance of a highly penetrant variant expected to be de novo for pathogenicity assumption was considered as a non-segregation and, therefore, a key step for benign or likely benign classification. Studies like ours may help to identify rare benign variants and improve the correct interpretation of genetic findings.

## Introduction

Genomic studies based on next-generation sequencing (NGS) technology are cost-effective alternatives to determine the molecular diagnosis of patients with rare diseases of monogenic etiology because they allow the concomitant study of several known genes associated with genetic conditions. On the other hand, these techniques may generate massive amounts of data and unravel several rare genomic variants, which brings challenges in the interpretation of these findings.

Variant classification is a systematic process that gathers different pieces of evidence from multiple sources, including scientific literature, control databases, in-silico predictors, among others, and aims a correct interpretation of genetic findings. The American College of Medical Genetics and Genomics in conjunction with the Association for Molecular Pathology (ACMG-AMP) created guidelines for the interpretation of sequence variants, becoming an important step toward a more uniform classification process. Among the recommendations, we highlight (1) the use of specific standard terminology: ‘pathogenic’, ‘likely pathogenic’, ‘uncertain significance’, ‘likely benign’, and ‘benign’ to describe variants identified in Mendelian disorders and (2) a standardized process for classification of variants based on criteria using typical types of variant evidence^1^. Among these types of evidence, segregation of a variant in an affected family may be evidence for pathogenicity; for several diseases, though, the non-inheritance (e.g., de novo event) is the expected mechanism for pathogenicity. On the other hand, lack of segregation of a variant with a phenotype may provide strong evidence against pathogenicity.

Another category recommended by ACMG-AMP is in silico analysis. Many in silico algorithms have been developed to predict the impact of missense variants and some of these tools have demonstrated a superior performance, such as REVEL [2. 3].

The finding of a variant of unknown significance (VUS) can be problematic for patients and clinicians working in clinical genetics setting^4–8^. Although definitive re-classification of a VUS as pathogenic or benign may eventually occur, the timeline is typically many years, and may be indefinite for rare VUS, especially if the disease is uncommon^5,9,10^. Therefore, efforts for the differentiation of benign and pathogenic variants gain importance.

In this article, we used segregation analysis and other molecular data to reclassify to benign or likely benign several rare clinically curated variants of autosomal dominant inheritance from a cohort of 500 Brazilian patients with rare diseases.

## Materials and methods

### Selection of cases

This study is focused on novel data (“Additional Findings”) from molecular findings obtained from exome sequencing (ES) analysis of 500 samples of adult, pediatric and fetal patients. Full details of the clinical features of patients, molecular analysis, bioinformatics protocols, clinical data, and molecular data for primary findings, secondary findings and carrier status for recessive diseases were previously published^10,11^. These samples were collected from 2016 to 2020 in facilities of the Fleury Group, which is one of the largest private diagnostic laboratories in Brazil.

This study has been performed in accordance with the Declaration of Helsinki, followed the best standards for scientific research and strictly followed Brazilian law for research involving human subjects. Written informed consent was obtained from all the participants. This study was granted ethics committee approval from Grupo Fleury and Faculdade de Medicina da Universidade de São Paulo (Plataforma Brasil; CAAE# 02617018.3.0000.5474; Fleury# 3.372.339).

Primary findings were reported when pathogenic or likely pathogenic variants were observed in a gene that was associated with the patient’s phenotype, with compatible zygosity and an adequate inheritance pattern; these cases were considered positive. Secondary findings (conditions unrelated to the indication for testing that might impact the health or quality of life of patients or other family members) were reported for pathogenic and likely pathogenic variants in the ACMG gene list and exceptionally in other genes determined to be clinically relevant by our team of specialists^12^. Full details of the primary and secondary findings can be found elsewhere^10^. The carrier status for pathogenic or likely pathogenic variants of autosomal recessive conditions has also been published elsewhere^11^.

The samples were collected from exclusively symptomatic patients who had undergone molecular investigation for suspected diseases of genetic etiology. The clinical data were collected through a comprehensive pretest form completed by the attending physician or family, medical reports and clinical notes provided to the laboratory.

### Molecular analysis and bioinformatics

Molecular analysis and bioinformatics protocols followed the exact workflow as published in previous works by our group^10,11^. In summary, DNA from the proband and both parents were extracted from peripheral blood leukocytes, saliva or prenatal samples of villus biopsy or amniotic fluid after appropriate cell culture. Exome capture used Agilent Clinical Research Exome v1 according to the manufacturer’s instructions and sequencing was performed using an Illumina NextSeq platform. Exome data were aligned to the GRCh37.75/hg19 reference genome using the Burrows–Wheeler Aligner (BWA; version 0.7.17-r1188). Variants (single-nucleotide variants [SNVs] and indels) were identified following the best practices of the Broad Institute using the Genome Analysis ToolKit (GATK, version 3.8-0-ge9d806836) software and annotated using Variant Effect Predictor (VEP, version 88.14). All exomes met a minimum of 95% of target bases covered at > 10 × . In-house bioinformatic pipelines were developed by a dedicated team of specialists. The mitochondrial genome and copy number variants were not studied.

### Variant selection and segregation study

The variant analysis workflow has also been performed according to our previous works^10,11^. In summary, at least two parallel analyses were performed to preselect variants considering (1) clinical relevance and overlap of gene harboring the variant and the provand’s manifestation, (2) allele frequency < 1%, (3) relevant functional impact, (4) relevant reports from databases (e.g., ClinVar and HGMD) and (5) previous reports from the literature. The first step of variant preselection consisted of filtering those already reported in ClinVar or HGMD as disease-causing and reclassifying them according to ACMG guidelines. Then, the same filtering-reclassifying approach would include (1) variants of predicted loss-of-function harbored by genes with evident sensitivity for haploinsufficiency and (2) rare missense variants harbored by genes with missense constraint. The last step in variant preselection included variants with allele frequency < 1% harbored by genes with clinical overlap with the proband’s phenotype.

These preselected variants were then discussed in a board comprising three experts. After this first board meeting, selected variants would undergo Sanger confirmation in the proband and inheritance pattern determination when the parental samples were available. Paternity and maternity were not confirmed by any specific test in our cohort, although nonpaternity/nonmaternity could be inferred by genotyping multiple rare variants. Parents were considered non-affected, unless otherwise reported in the clinical notes provided to the laboratory.

The samples were analyzed by three distinct protocols as follows^10,11^: Protocol 1 (cases 1–289; n = 289), in which the samples were processed in our laboratory as described above and variants were interpreted using an in-house web interface (GTAC); Protocol 2 (cases 290–387; n = 98), in which the samples were processed in our laboratory as described above and variants were analyzed using a commercially available diagnostic decision support platform by Emedgene Technologies LTD (Tel-Aviv, Israel) (www.emedgene.com); Protocol 3 (cases 388–500; n = 113), in which the samples were sent to Centogene AG (Rostock, Germany) (www.centogene.com) and processed according to the protocols of this third-party laboratory, including DNA extraction, bioinformatic pipeline, CNV analysis, and variant analysis. For Protocol 3, the primary findings reported by Centogene were confirmed by in-house Sanger sequencing of the proband and genitors to determine the inheritance pattern.

### Additional findings—rare clinically curated variants

Variants preselected as described above in the board of experts were preselected because they presented relevantly low allele frequencies (< 1%) and potential relevance to the clinical manifestation of the corresponding patient. All variants were clinically suspected as the causative etiology for all patients and were harbored by genes associated with highly penetrant conditions of autosomal dominant inheritance. As a de novo event was expected for pathogenicity assumptions, these variants underwent Sanger confirmation in the proband and inheritance pattern determination when the parental samples were available. Individual data for each case, along with the molecular data and segregation studies for all variants can be accessed in the Supplementary Material.

### Variant classification protocol

All variants were reviewed and reclassified for the purpose of this article. The complete details, nomenclature and classification information are available in the Supplementary Material. Variants were classified according to ACMG guidelines^1^ with assistance of the third-party ACMG calculator Varstation ® (VarsOMICS®, São Paulo, Brazil; www.varsomics.com). Internal adaptations of ACMG guidelines used in this work may be found below.

### Internal adaptations of ACMG guidelines

ACMG presents specific rules to classify variants^1^. Each benign criterion is weighted as stand-alone (BA1), strong (BS1–4), or supporting (BP1–6). Benign variants need either a stand-alone (BA1) criterion or two strong (BS1–4), while likely benign variants need one strong and one supporting criterion or at least two supporting criteria. Although variant classification followed the ACMG guidelines, some adaptations were adopted especially for benign criteria BS4, BS1, BS2, BP4 and BP5:

BS4—Variant does not segregate with the disease: considering that the lack of segregation of a variant with a phenotype provides evidence against pathogenicity, this criterion was used if a variant expected to be de novo was inherited from unaffected parents in genotype-positive and phenotype-negative scenarios. If the gene harboring the variant is associated only with early-onset conditions, the non-segregation was considered a strong evidence of non-pathogenicity (BS4_strong), while if the gene is associated with later-onset manifestations the non-segregation was considered a supporting evidence (BS4_supporting). All conditions analyzed in this study were considered enough penetrant for the application of segregation criteria.

BP5 —Variant found in a case with an alternate molecular cause for the disease. When a variant was observed in a case with a clear alternate genetic cause of disease (i.e., variant in a case with an alternate “Primary Finding”) for which the reported variant is unlikely to contribute or modulate expressivity of the primary finding, this was considered supporting evidence to classify the variant as benign (BP5_supporting). Primary finding has already been defined above.

BS1—Allele frequency is greater than expected for disorder: we have used a conservative approach to use this criterion: variants received a BS1_strong only when presented with a frequency greater than 0.1% for autosomal dominant diseases in the gnomAD database.

BS2—Observed in a healthy adult individual for a recessive (homozygous), dominant (heterozygous), or X-linked (hemizygous) disorder, with full penetrance expected at an early age: since the objective of our study is to analyze the impact of variants exclusively in the autosomal dominant inheritance pattern, we have used a threshold to apply BS2_strong of 5 individuals harboring the same variant in the gnomAD database.

PM2_supporting-  Absent from controls for AD in gnomAD. We adjusted all PM2 criteria to PM2_supporting in agreement with recent recommendations from the Clingen working group.

PP3-pathogenic for REVEL score > 0.7, according to the recommendations for best practices of United Kingdom Association for Clinical Genomic Science^13^.

BP4- REVEL score < 0.4, according to the recommendations for best practices of United Kingdom Association for Clinical Genomic Science^13^.

### Genes, diseases, inheritance and penetrance

Information about genes and their corresponding diseases along with inheritance mechanism and penetrance was recalled from The Clinical Genome Database website (https://research.nhgri.nih.gov/CGD/search; date of access: June, 2021) of the National Human Genome Research Institute (NHGRI), based in the United States^14^.

### Ethics approval

This study has been performed in accordance with the Declaration of Helsinki. This study was granted ethics committee approval from Grupo Fleury and Faculdade de Medicina da Universidade de São Paulo (Plataforma Brasil; CAAE# 02617018.3.0000.5474; Fleury# 3.372.339).

### Consent to participate

Written informed consent was obtained from all the participants.

### Consent for publication

All authors are aware, consented and approved this publication.

## Results

We have analyzed a total of 334 occurrences of 327 unique heterozygous variants (seven variants recurred twice) in 210 different genes.

Regarding the 210 different genes, 171 (81.4%) are solely associated with autosomal dominant diseases and this group of genes harbors 273 occurrences of 267 unique variants (81.7%). Another 37 genes (17.6%) are associated with autosomal dominant and autosomal recessive forms and this group harbors 59 occurrences of 58 unique variants (17.7%). Two genes (*SCNN1A* and *GPR98*; 1%) are associated with either autosomal dominant, autosomal recessive or digenic inheritances and harbor two occurrences of two unique variants (0.6%).

Still regarding the 210 different genes, 90 genes (42.9%) are associated with exclusive pediatric-onset conditions and harbor 145 occurrences of 141 unique variants (43.1%): 138 variants were inherited from asymptomatic parents and received BS4 (strong) criterion, while two variants (*GRIN2A* and *SCN1B* genes, both variants harbored by the same patient 274) were not found in both parents and in one case paternal sample was not available (*SCN1A* gene, case 47). Six genes (2.9%) that are associated with adult-onset conditions harbor eight occurrences of seven unique variants (2.1%), to which a BS4_supporting criterion was attributed. Finally, 114 genes (54.3%) that harbor 181 occurrences of 179 unique variants (54.7%) were not classified by NHGRI regarding the onset of the correspondent diseases: 176 variants were inherited from asymptomatic parents and were attributed the BS4_supporting criterion; two variants were not found in both parents (*GATAD2B* and *CIC* genes, cases 38 and 174 respectively) and in one case paternal sample was not available (*KCNC3* gene, case 109).

All 334 occurrences of 327 unique heterozygous variants were confirmed by Sanger sequencing in probands. Additionally, parental segregation studies were performed for both parents in 332 cases and paternal samples were not available for two cases (cases 47 and 109). Variants were inherited from mothers in 168 cases (50.3%); inherited from fathers in 158 (47.3%); were not found in both parents in four (1.2%); paternal sample was not available in 2 cases (0.6%) and in two other cases both parents were heterozygous for the same variant (0.6%).

Regarding the 327 unique variants, 139 (42.5%) were not found in the gnomAD database (gnomad211) and were attributed the PM2_supporting criterion; 87 variants (26.6%) presented from one and less than five controls in gnomAD. Another 100 variants (30.6%) were present in five or more controls in gnomAD and were attributed the BS2 criterion. Among these 100 variants, 11 (3.4%) presented a frequency higher than 0.1% in gnomAD and also attributed the BS1 criterion.

For 81 unique variants (24.8%), a clear alternate genetic cause for the patients’ conditions was unraveled by ES. For these variants, BP5 criterion was used. All alternate causes for diseases (primary findings) were published elsewhere^10^.

**Figure 1:**
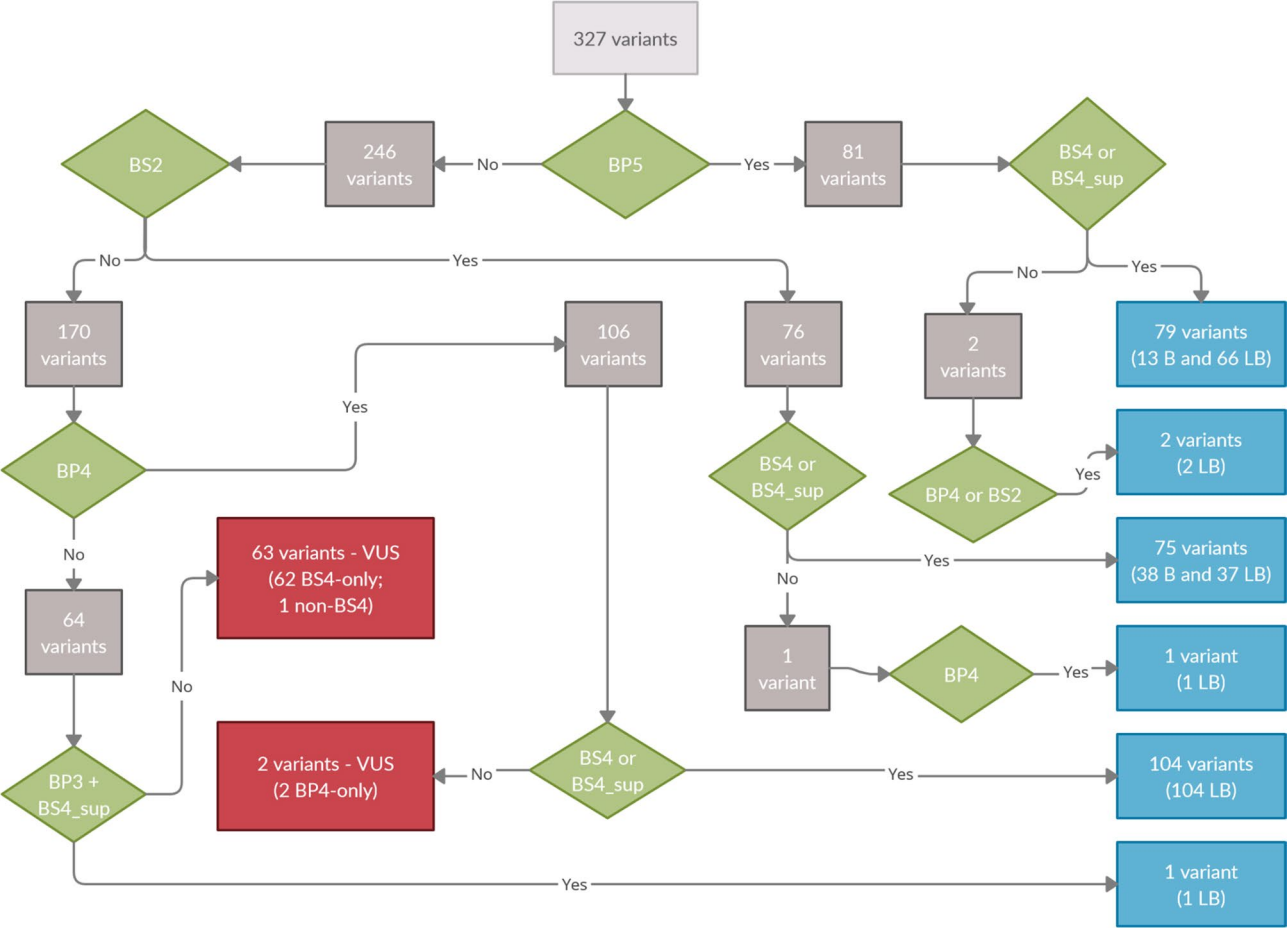
Workflow of classification of all 327 variants. The figure shows the workflow with the key benign criteria used to classify all 327 unique rare variants in the autosomal dominant model of inheritance. Green diamonds represent the main benign criteria used for variant classification; grey squares represent the number of variants flowing in the decision tree; blue squares congregate all variants classified as benign or likely benign and red squares represent the variants that did not meet minimum requirements for classification as benign or likely benign and kept a final classification of variants of unknown significance (VUS). Note that the 327 variants are initially divided into two groups: 81 variants received the BP5 (supporting) criterion because these variants occurred in patients with a clear molecular alternate cause of disease; 79 out of these 81 variants were inherited from predictively asymptomatic parents, received either BS4 or BS4_supporting criterion and were classified as benign (13 variants) or likely benign (66 variants); two out of these 79 were not inherited (one variant was not found in both parents and for the other one paternal sample was not available): one of them received the BP4 criterion and the other received both BS2 and BP4 criteria and both were classified as likely benign. The remaining 246 variants did not receive BP5 and followed the classification flow. Among the remaining 246 variants, 76 received BS2 criterion because they were found in at least five controls from gnomAD database: 75 were inherited from parents and received an additional BS4 or BS4_supporting and were classified as benign (38 variants) or likely benign (37 variants); one variant was not inherited (not found in parental samples), but as it received the BP4 criterion it was classified as likely benign. Among the remaining 170 that did not receive either BP5 and BS2 criteria, 106 were predicted as having benign effect by REVEL and received BP4 criterion: 104 variants were inherited from parents, received additional BS4 or BS4_supporting criteria and were all classified as likely benign; two variants were not found in parental samples and were classified as VUS since they received only BP4 criterion. Finally, 64 variants did not receive BP5, BS2 nor BP4 criteria: one of them was an inframe indel in non-functional domain (BP3), inherited from the mother (BS4_supporting) and was classified as likely benign; 63 variants did not meet minimum requirements for reclassification (62 were inherited and received only the BS4 or BS4_suporting criteria and one variant was not inherited) and were considered VUS.

**Figure 2:**
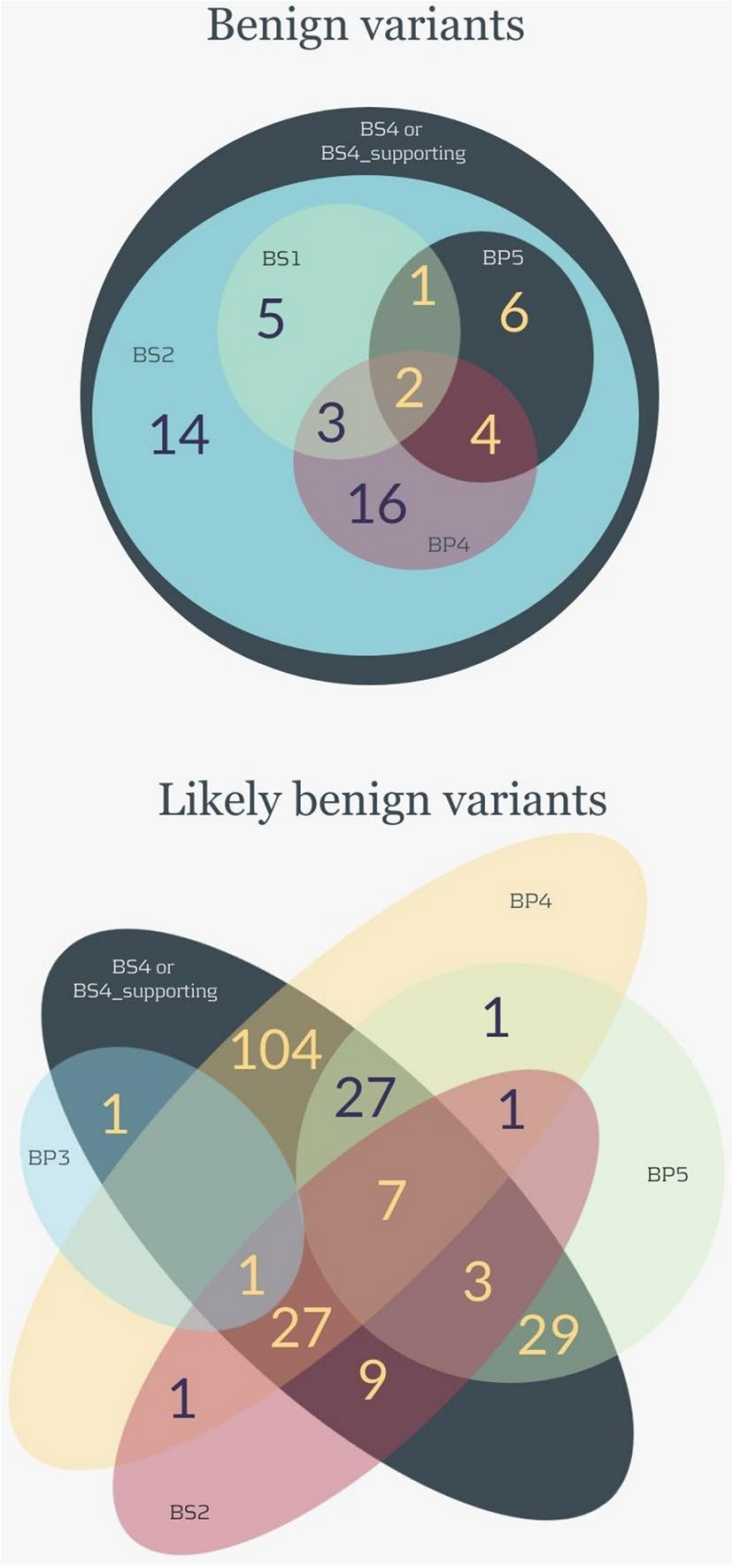
Venn diagrams of benign and likely benign variants. The figure shows the distribution of all benign criteria used in this work to classify 51 variants as benign (upper diagram) and 211 variants as likely benign (lower diagram).

Figure 1 resumes the workflow of classification and shows the key benign criteria used to classify all 327 unique rare variants in the autosomal dominant model of inheritance. Regarding the ACMG classification, 51 unique variants (15.6%) were classified as benign, among which 47 are missense, one nonsense (stop gain) and three in-frame indels. Additionally, 211 unique variants (64.5%) were classified as likely benign: four stop gains, one start loss, one frameshift-predicted indel, four in-frame indels, two canonical splice-site alterations and the remaining 199 missense variants. Figure 2 contains Venn diagrams of benign and likely benign variants showing the distribution of all benign criteria used in this work.

Considering all variants classified as benign or likely benign (n = 262; 80.1%), we did not find dbSNP correspondence for 76 variants (29%) and ClinVar entrances were not found for 189 variants (72.1%). ClinVar entrances were available for 73 variants (27.9%), among which 15 were classified as benign or likely benign, 40 as VUS, one as likely pathogenic and for 17 variants ClinVar classification was conflicting among participating laboratories.

The remaining 65 unique variants (19.9%) were classified as variants of unknown significance. Two thirds of these variants (n = 44) were not found in the gnomAD database.

For unique variants harbored by genes associated with pediatric-onset conditions, 73% (103/141) were classified as benign or likely benign, while for variants harbored by genes associated with adult-onset conditions, 71.4% (5/7) were classified as likely benign. As for variants harbored by genes non-classified by NHGRI, 86% (154/179) were classified as benign or likely benign.

## Discussion

We have used segregation analysis and other molecular data to reclassify rare variants to benign (n = 51) or likely benign (n = 211) in a Brazilian cohort of rare diseases. All variants are harbored by genes associated with autosomal dominant disorders. Proper interpretation of rare variants is a crucial step for adequate molecular diagnosis and, consequently, clinical management and genetic counseling. Therefore, efforts to distinguish pathogenic variants from rare benign variants are a key step in molecular diagnosis, but ascertaining which rare variants have clinical/health impact remains a major challenge.

We have used a systematic approach to preselect the variants in this study. Besides a low allele frequency in control databases, reports from literature and functional impact, all variants were clinically curated: they were harbored by genes associated with autosomal dominant disorders similar to all patients’ manifestations. In other words, all variants were associated with autosomal dominant disorders and were judged highly suspicious to explain the final diagnosis that had led to exome sequencing.

Segregation study was assessed to investigate the clinical relevance of the rare variants found in our cohort because a de novo event was expected for all 327 suspicious variants. Segregation is important to assess the pathogenicity of a variant. Indeed, variant segregation in patients and corresponding families are widely used for novel disease-associated genes and pathogenic/likely pathogenic variants discoveries. In this study, we have used the same approach for the opposite reason: uncover benign/likely benign variants. The inheritance of a variant expected to be de novo for pathogenicity assumption was considered in our study as a non-segregation and, therefore, a key step for benign or likely benign classification. However, this was not the sole step in this process, as discussed below.

Figure 1 resumes the key workflow steps of the variant classification. A total of 81 variants occurred in patients with a clear molecular alternate cause of disease and received the BP5 (supporting) criterion. All these 81 variants were classified as benign (n = 13) or likely benign (n = 66) because they also received, at least, another benign criterion either because were inherited from predictively asymptomatic parents (n = 79) receiving BS4 or BS4_supporting criterion or REVEL score suggested no impact (n = 2). Note that two variants were not inherited—non-inherited variants will be discussed below. In this branch of cases shown in Fig. 1, the combination of BP5 and BS4/BS4_supporting was key for non-pathogenicity assumption. Figure 2 demonstrates that this combination of criteria happened for 13 benign variants and 66 likely benign variants: 29 likely benign variants received exclusively these two criteria and this combination was critical for final classification, while the remaining variants received other benign criteria.

Another key step in the classification is the frequency in the control database. In this study, we used only the gnomAD database, a fact that may have underestimated the “benign weight” of BS2 criterion because other databases might suggest higher frequencies especially for ethnicities not covered by gnomAD; on the other hand, the quality of variant calls and integrity of database might be an uncontrolled bias. All variants found in five or more controls in gnomAD were eventually classified as benign or likely benign because they received other benign criteria. Regarding the benign variants, 14 received exclusively the combination BS4 (variants inherited from parents and associated with conditions curated as pediatric onset) and BS2. As for the likely benign variants, nine variants received exclusively BS4_suporting (variants inherited from parents and associated with conditions not curated by NHGRI) and BS2.

A fourth key step was in-silico prediction. In this study, we have used REVEL, which is an ensemble method for predicting the pathogenicity of rare missense variants, as our sole in-silico predictor. REVEL has shown better performance when compared to other methods especially for rare neutral variants^2,3,15^. These new pieces of evidence demonstrate adequate strength of REVEL as a benignity predictor and make this tool fit adequately in our study, which aims to unravel rare neutral variants. The limited use for missense variants, though, is a limitation of REVEL approach.

Among the 170 that did not receive either BP5 and BS2 criteria, 106 were predicted as having benign effect by REVEL (score < 0.4) and received BP4 criterion; 104 of these variants were inherited from parents (received BS4 or BS4_supporting) and the combination of BP4 + BS4/BS4_supporting was critical for the final classification as likely benign.

Finally, 65 variants did not meet minimum requirements for reclassification as benign or likely benign and were considered VUS, among which 63 were inherited from a parent (received solely BS4 or BS4_supporting).

A total of six unique variants were not confirmed to be inherited from either parent in our study, among which four were not found in parental samples (a supposed de novo event) and for the remaining two, the paternal samples were not available, though these two variants were not found in the maternal samples. Half of these variants (n = 3) were classified as likely benign considering other molecular data. Although it is widely known that several de novo genome changes can be associated with a multitude of pathological conditions, a broad range of de novo molecular events is also characteristic for individuals obtained from a general human population^16^.

For the majority of variants classified as benign or likely benign, we did not find a ClinVar classification (n = 189; 72.1%). Even for variants with ClinVar entrances, we found a concordance of classification with our study only for 15 variants.

Our study approach focuses on variant segregation with phenotype and makes several simplifying assumptions, including (1) parents were asymptomatic: we recognize that several dominant diseases may present varying degrees of severity and it may be difficult for clinicians to identify oligosymptomatic patients; (2) non-segregation was considered a strong benign criterion for child-onset diseases and a supporting criterion for later-onset diseases or onsets non-classified solely by NHGRI: NHGRI database lacks information for several genes, including some widely known early-onset genes (e.g., *ASH1L, KMT2A, DEAF1, TRPV4, CHD2, SRCAP, ANKRD11, RAI1, ZEB2, SHANK3, ARID1B*, and others) and this fact may have underestimated non-segregation (unclassified genes received a BS4_supporting); (3) variants associated with adult-onset conditions were a minority (seven unique variants) and segregation analysis for these cases may be more complex to understand: we opted to apply a BS4_supporting criterion considering that parents were asymptomatic and we did not have information of other affected family members, even though we may have overestimated the non-segregation effect for these seven variants; (4) our method applies for genetic sequence variants of high penetrance and dominant inheritance: for this model, we considered all conditions sufficiently penetrant for segregation/non-segregation purposes.

The model that we have used presents limitations for more complex inheritance, including unknown codominant, digenic or even oligogenic mechanisms or for recessive inheritance. It is noteworthy that some variants are harbored by genes associated with both autosomal dominant and autosomal recessive forms. However, all variants were clinically selected because only the autosomal dominant form of the corresponding disease was similar to patients’ phenotypes and a de novo event was expected for pathogenicity assumption. Therefore, the non-segregation of these variants for the autosomal dominant model was considered relevant for non-pathogenicity.

It is important to note, though, that there are growing pieces of evidence that rare variants make important contributions to human phenotypic variation and disease susceptibility, though detecting the effects of rare variants in complex traits is challenging because 1) it generally requires very large sample sizes to achieve statistical power and 2) rare SNVs are population-specific, which implies difficulties for replication of disease associations across different populations^17^. Therefore, it is possible (or even likely) that a rare variant classified as B/LB for autosomal dominant Mendelian trait may have health/disease implications for complex traits, for recessive forms or even for unknown recessive forms of a gene.

Our study presents important limitations. First, it analyzed the impact of single variants in monogenic-based models of autosomal dominant diseases and did not consider more complex interactions that might modulate phenotypes. Another limitation is that several variants were classified as likely benign/benign based solely on ACMG criteria but were not definitively proven to be benign. We took several precautions to apply some ACMG benign criteria that may have underestimated their effects: BS2 required at least five controls from gnomAD, BS1 required a frequency of at least 0.1% in gnomAD (a frequency considered high for many rare conditions). Even though we followed the ACMG criteria for variant classification, we may have falsely classified some variants as B/LB since we relied solely on presumptively assuming mechanisms without proper functional studies.

Even taking extra precautions that underestimate the weight of several benign criteria (e.g., BS2, BS1) and underestimating the non-segregation for several conditions non-classified by NHGRI, the majority of variants (n = 262; 80.1%) were reclassified as benign or likely benign. One main reason for this is that it is a lot easier for a variant to be classified as B/LB than pathogenic or likely pathogenic because only two supporting benign criteria are enough for LB classification. We used the term “easier” for two reasons: easier because it requires less criteria for B/LB classification and easier because they do not have up- or -downgrades such as several pathogenic criteria. These assumptions have made us raise concerns regarding the wide application of benign criteria. They also made us unilaterally downgrade BS4 criterion for later-onset or non-classified-onset conditions. On the other hand, the addition of extra supporting benign criteria does not influence in the final classification: for instance, a variant receiving a combination of BS2 and BP4 will have the same likely benign classification of a variant receiving BS2, BS4_supporting, BP4 and BP5, though the likelihood for this later variant to be benign is greater than the former.

We did not find comprehensive studies that address segregation analysis and classification of B/LB variants in Brazil or any other Latin American countries. These countries are generally underrepresented populations in international databases and genetically heterogeneous with important genetic contributions from Amerindians, African-Americans and Western-Europeans. The more NGS and segregation studies become available in such nations, the more will be known of the rare regional benign variants.

Literature presents an important limitation regarding the description of benign/likely benign variants because studies and research journals generally present a strong bias for positive results. We believe that studies like ours are valuable for identifying rare benign variants and this strategy may improve the correct interpretation of genetic findings.

## Supplementary Information

Supplementary Information.

## Data Availability

All data are provided as “Supplementary Material”.
